# Evaluation of the hemostatic effect of a combination of hemostatic agents and fibrin glue in a rabbit venous hemorrhage model

**DOI:** 10.1186/s12883-021-02272-y

**Published:** 2021-07-07

**Authors:** Katsumi Takizawa, Daiki Okazaki, Yoshitaka Takegawa, Yuki Koga, Masataka Sagata, Kenichi Michishita, Noriko Shinya

**Affiliations:** 1 Department of neurosurgery, Japanese Red Cross Asahikawa Hospital, Asahikawa-shi, Hokkaido Japan; 2 Non-Clinical Study Department, Non-Clinical Development Section, KM Biologics Co., Ltd, Kumamoto-shi, Kumamoto, Japan; 3 Medical Affairs Section, Research & Development Division, KM Biologics Co., Ltd, 1-6-1 Okubo, Kita-ku, Kumamoto-shi, , Kumamoto, Japan; 4 Development Planning & Coordination Section, R&D Coordination & Administration Department, KM Biologics Co., Ltd, Kumamoto-shi, Kumamoto, Japan

**Keywords:** Fibrin glue, Hemostasis, Gelatin sponge, Animal model

## Abstract

**Background:**

In neurosurgery, it is important to use local hemostatic agents. We have explored a more powerful method of hemostasis by the combination of commercially available hemostatic agents with fibrin glue in the hopes of synergistic effects.

**Method:**

A bleeding model was constructed by puncturing the rabbit posterior vena cava with a needle. After applying the sample to the bleeding point, compression was performed for 10 s. If temporary hemostasis was achieved after pressure release, a 30 s wash was performed to confirm that ultimate hemostasis was achieved. Up to three hemostasis attempts were performed on the same bleeding point until hemostasis was achieved, and the number of attempts required for hemostasis was counted. If hemostasis was not achieved after three attempts, it was counted as four times. Four groups were evaluated: (1) gelatin sponge alone, (2) gelatin sponge + fibrin glue, (3) oxidized cellulose alone, and (4) oxidized cellulose + fibrin glue; each group was tested 16 times.

**Results:**

The median value (range minimum value–maximum value) of the number of hemostatic attempts in Group 1 to Group 4 was 3 (1–4), 1 (1–1), 4 (4–4), and 4 (2–4). In Group 2, there were two test exclusions owing to deviations of the test procedure.

**Conclusions:**

The compatibility of gelatin sponge and fibrin glue was very good, with a very strong and rapid hemostatic effect compared to other methods, showed its usefulness. This combination method may be effective for a variety of venous hemorrhages in neurosurgery.

**Supplementary Information:**

The online version contains supplementary material available at 10.1186/s12883-021-02272-y.

## Background

Hemostasis occupies an important place in all operations, and the appropriate hemostatic procedure varies according to the bleeding site. Hemostatic procedures include compression, suturing, ligation, and electrocautery. However, in neurosurgery, there are many situations in which hemostasis using a topical hemostatic agent is best because of concerns about aggravation of neurological symptoms owing to vascular occlusion and the effects on the surrounding brain tissue. Various products that have different characteristics are commercially available as topical hemostatic agents that can be used in neurosurgery [1–4]. Achieving more rapid and reliable hemostasis ensures that the operative field is clean and that subsequent surgical manipulation can proceed smoothly. Selection of an effective topical hemostatic agent is crucial to reduce the operating time and prevent delayed hemorrhagic complications. We hypothesized that the combination of a topical hemostatic agent and fibrin glue would provide a synergistic effect. Several combinations have been reported[5–11], but the optimal combination for hemostasis is controversial. We explored the combination of commercial hemostatic agents and fibrin glue that would produce the most potent hemostatic effect in a synergistic manner.

## Methods

### Animals

Rabbits (Japanese white, 6 weeks old, 2.5–3.0 kg, male) were purchased from KITAYAMA LABES CO.,LTD. (Nagano, Japan) and used with a 1-week acclimation period in individual cages. For induction of anesthesia, 5 mg/kg (0.25 mL/kg) of xylazine was administered intramuscularly, and 35 mg/kg (0.7 mL/kg) of ketamine was administered intramuscularly approximately 5 min later. Anesthesia was maintained by ear vein infusion (50 mL/h) of Ringer’s lactate solution with 4 % v/v ketamine. Rabbits were euthanized at the end of the experiment by blood liberation by cutting the abdominal aorta while maintaining anesthetic depth.

### Materials

For topical hemostatic agents, gelatin sponge (Gelfoam®, Pfizer Inc., New York, NY) and oxidized cellulose (SURGICEL® Nu-Knit Absorbable Hemostat, Ethicon Inc., New Brunswick, New Jersey), which are commonly used in neurosurgery, were selected. These two topical hemostatic agents were used alone or in combination with fibrin glue (BOLHEAL®, KM Biologics Co., Ltd., Kumamoto, Japan) and the results were compared in a total of four groups.Group 1 is gelatin sponge alone, Group 2 is combination of gelatin sponge and fibrin glue, Group 3 is oxidized cellulose alone, and Group 4 is combination of oxidized cellulose and fibrin glue. Fibrin glue consists of two fluids, a fibrinogen solution and a thrombin solution, and is a biological adhesive that gels quickly when mixed.

### Procedures

As a rabbit venous bleeding model, rabbits were operated on under anesthesia and, after exposure of the posterior vena cava, bleeding was induced by direct puncture of only the upper wall of the posterior vena cava with a 19G needle. Procedure was performed with neurosurgical pads (Bemsheets™, Kawamoto Corporation, Osaka, Japan) and an irrigation/suction device (Kamiyama Irrigation Suction Version I, Ohwa Tsusho Co., Ltd., Tokyo, Japan) using saline. After confirming the bleeding point while controlling the bleeding, the sample was securely applied to the bleeding point and compressed and fixed for 10 s. Achievement of hemostasis was assessed macroscopically and signs of slight bleeding were considered a failure. If no bleeding was observed after the 10 s hemostatic procedure, a 30 s wash water pressure load was added using the irrigation/suction device, and if no bleeding was observed after application of hydraulic pressure, hemostasis was judged to be achieved. If hemostasis was not achieved, the applied hemostatic sample was completely removed, a new hemostatic sample in the same group was used, and hemostasis was attempted again in the same manner. Hemostasis was attempted up to three times per bleeding point, and the number of attempts required to achieve hemostasis was counted. If hemostasis was not achieved after three hemostatic attempts, it was counted as “4”. Deviations in hemostasis procedures were excluded from the evaluation.

Groups 1 and 2 used gelatin sponges and Groups 3 and 4 used oxidized cellulose as samples. Groups 1 and 3 used 3 mm square trimmed hemostatic agents as intact samples. In groups 2 and 4, hemostatic agents trimmed to 3 mm square were soaked in 10 µL of fibrinogen solution immediately prior to use, applied to the bleeding point, and adhered to the bleeding point by dripping an appropriate amount of thrombin solution onto the sample and gelling. Additional movie files show this in more detail (see Additional file 1, 2).

In the posterior vena cava, four bleeding points were created and one test for each of the four groups was performed at each bleeding point to eliminate individual variability. The bleeding points were created at least 2 cm apart to avoid the effects of each test. In addition, the bleeding point and the group used for hemostasis were switched from one animal to another, and the number of applications to the distal and central sides of the blood vessels in each group was allocated equally. In determining the number of samples, it was necessary to conduct one set of four tests to ensure uniform allocation. To ensure reproducibility, this set was repeated three times. Sixteen rabbits were used. Sixteen tests were conducted for each group. Surgical procedures were performed using a laparoscopic endoscope (LTF-S-190-10, Olympus Corporation, Tokyo, Japan) with an enlarged field of view. This procedure was performed by a single expert.

### Statistical analysis

Median calculations and graphs were performed using EZR (Saitama Medical Center, Jichi Medical University Site, Saitama, Japan). EZR is a graphical user interface for R (The R Foundation for Statistical Computing, Vienna, Austria, version 2.13.0) [12].

## Results

**Fig. 1:**
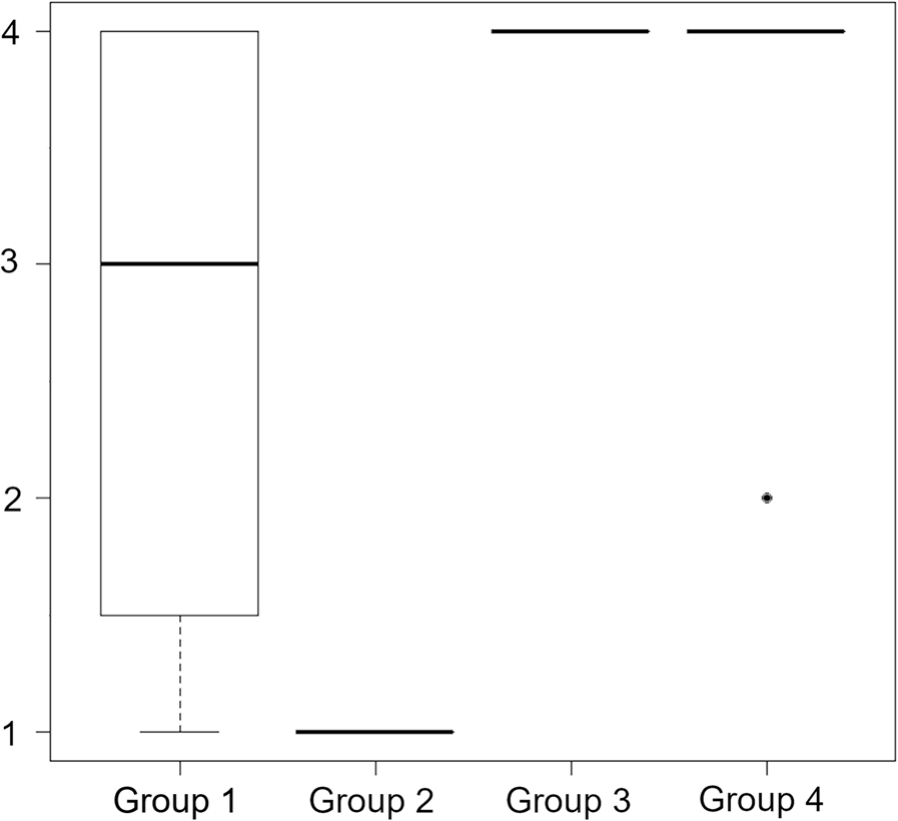
Box-and-whisker plot of the number of hemostatic attempts in each group (*N* = 16 per group. In Group 2, two tests were excluded from the study results because of deviations from the study procedures)

The results are presented in Table 1; Fig. 1. The median number of hemostatic attempts in each group was 3 (1–4) in Group 1, 1 (1–1) in Group 2, 4 (4–4) in Group 3, and 4 (2–4) in Group 4. In Group 2, two tests were excluded from the study results because of deviations from the study procedures. In one case, the time required for compression was a few seconds longer than that specified because of the adhesion of the forceps to the sample; the forceps could not be removed from it. In another, after slight bleeding in the first application, hemostasis was achieved on additional compression with the sample held at the bleeding point to temporarily control bleeding until the second sample was applied. Although we attempted to remove the hemostatic sample, the sample was firmly adhered to the blood vessel, and the second application was not performed because of concerns of dilatation of the needle hole caused by its removal. Incidentally, complete hemostasis was achieved in the first sample in both cases.

**Table 1:** Results of the Number of Hemostatic Attempts in Each Group

Rabbit	Group 1	Group 2	Group 3	Group 4
**1**	2	1	4	4
**2**	4	1	4	4
**3**	1	1	4	4
**4**	3	1	4	4
**5**	2	1	4	4
**6**	4	-^a^	4	4
**7**	3	1	4	4
**8**	3	1	4	2
**9**	2	-^a^	4	4
**10**	4	1	4	4
**11**	1	1	4	4
**12**	1	1	4	4
**13**	4	1	4	4
**14**	4	1	4	4
**15**	1	1	4	4
**16**	4	1	4	4
**Median**	3	1	4	4
**Maximum**	4	1	4	4
**Minimum**	1	1	4	2

^a^ Excluded from the results because of deviation from the test procedure

## Discussion

The combination method of gelatin sponge and fibrin glue has been used for a long time in our institution and has been reported as a method of arachnoid repair [13]. In this article, it was used for hemostasis. This method is considered unique in that it can form a fibrin gel independent of the fibrinogen in the patient’s blood, unlike commercial products that interact with the fibrinogen in the patient’s blood to form a clot, such as Gelfoam Plus® Heamostasis Kit (Baxter International Inc., Deerfield, IL) that combines the gelatin sponge and thrombin or FLOSEAL© hemostatic matrix (Baxter International Inc.) and SURGIFLO® hemostatic matrix (Ethicon Inc.) that combines the gelatin foam and thrombin.

**Fig. 2:**
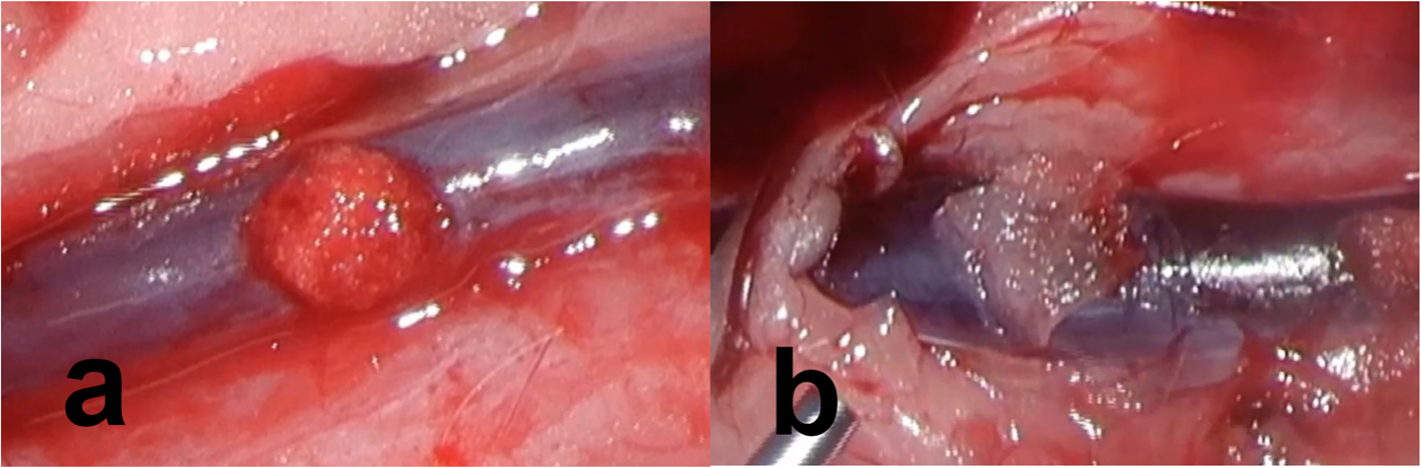
Actual visual field after hemostasis. **a**: In Group 2, the hemostatic agents adhered to the curve of blood vessels. **b**: In Group 4, adhesion along the vessel wall was not achieved, and the ends were elevated against the vascular curvature

In this study, gelatin sponge and oxidized cellulose were used alone or in combination with fibrin glue, and the hemostatic effect was compared among the four groups and the combination of gelatin sponge and fibrin glue tended to have the greatest hemostatic effect. The difference in the effect of gelatin sponge and oxidized cellulose in combination with fibrin glue was first thought to be because immediate denaturing of thrombin and fibrinogen caused by lowering of pH by oxidized cellulose [14]. In addition, when oxidized cellulose saturated with blood, it swells into a gelatinous mass that aids the formation of a clot, serving as a hemostatic adjunct in the control of local hemorrhage [15]. However, this gelatinization also occurred when the oxidized cellulose was soaked in the fibrinogen solution, which may have resulted in a loss of flexibility. In terms of flexibility, macroscopic observations after hemostasis revealed that the hemostatic agents were closely adherent to the vascular surface in Group 2 (Fig. 2 A, Additional file 1), whereas the ends of the hemostatic agents were elevated against the vascular curvature in Group 4 (Fig. 2B, Additional file 2). This loss of flexibility may have had a significant impact on the results of this study. There were 47 hemostasis applications of Group 4 throughout this study, of which 46 had failed hemostasis. Of the 46 failed attempts, 21 had achieved temporary hemostasis with the first 10 s of compression. However, bleeding occurred when the wash water pressure load was applied for the subsequent 30 s. The cause of this hemorrhage was thought to be the peeling off the sample due to the water pressure in the gap between the sample that had floated from the blood vessel and the tension caused by touching the perivascular tissue.

In contrast, the gelatin sponges used in Group 2 achieved a good fit with the blood vessels and the sample did not peel off either under wash water pressure loading during washing. If hemostasis was not achieved in Group 1, 3, and 4 tests, hemostasis was performed by the method of Group 2 to allow progression to the next test, and the subsequent test was performed. A total of 37 hemostatic procedures were able to achieve complete hemostasis using the method of Group 2 (data not shown). In all rabbits, no rebleeding occurred until sacrifice, and the strong adhesion and good fit to the blood vessels of the gelatin sponge in Group 2 was maintained. The authors believe that the fit between the hemostatic agents and the blood vessel is very important. The commercially available gelatin sponges are Gelfoam® and Spongel® (Astellas Pharma Inc., Tokyo, Japan), but because the Spongel® is rigid, we choose Gelfoam® to combine with fibrin glue.

There are four tips for using this method for venous bleeding: (1) to maintain a dry field of view using suction and neurosurgical pads and to accurately identify the bleeding points; (2) to ensure that the gelatin sponge soaked in fibrinogen solution is placed at the bleeding point and the bleeding is controlled at the point of compression; (3) to allow the thrombin solution to drip down into the fibrin gel in a dry state with no blood around it; and (4) to slowly and carefully withdraw the forceps used for compression after forming the fibrin gel.

Care should be taken when choosing this method for arterial bleeding. The author has experienced a case of delayed bleeding after surgery, although this technique has been used for arterial bleeding and achieved hemostasis in many patients. The delayed bleeding was a case of internal carotid-anterior choroid artery bifurcation aneurysm in which hemorrhage from the anterior choroid artery during its dissection from the aneurysm it was adhered to was stopped by this method. Hemostasis was achieved during surgery, but delayed bleeding occurred one week after surgery. After reoperation, it was confirmed that the bleeding had started at the site where hemostasis was performed by this method, and hemostasis was performed by suturing. When this method is indicated for arterial bleeding, the size of the artery and the degree of damage should be considered, but hemostasis by other methods may be considered in principle.

It is noteworthy that the Group 2 method had a high rate of hemostasis after only 10 s of application. Selection of a strong hemostatic method greatly reduces the operative time and prevents postoperative rebleeding. This nonclinical study suggests the usefulness of a combination of gelatin sponge and fibrin glue. In the future, we would like to consider demonstrating the usefulness of this method in nonclinical studies using confirmatory methods and in clinical studies.

## Conclusions

The compatibility of gelatin sponge and fibrin glue was very good, with a very strong and rapid hemostatic effect compared to other methods, showed its usefulness. This combination method may be effective for a variety of venous hemorrhages in neurosurgery.

## Supplementary Information

**Additional file 1: **Video 1. Group 2 (gelatin sponge + fibrin glue) technique.

**Additional file 2: **Video 2. Group 4 (oxidized cellulose + fibrin glue) technique.

## Data Availability

All data analyzed during this study are included in this published article.
